# A Four-Year Monocentric Study of the Complications of Third Molars Extractions under General Anesthesia: About 2112 Patients

**DOI:** 10.1155/2013/763837

**Published:** 2013-09-11

**Authors:** A. Guerrouani, T. Zeinoun, C. Vervaet, W. Legrand

**Affiliations:** ^1^Department of Oral and Maxillofacial Surgery, CHU Ambroise Paré University Hospital, Boulevard of Kennedy, 7000 Mons, Belgium; ^2^Department of Oral and Maxillofacial Surgery, School of Dentistry, Lebanese University, Hadath Campus, Beirut, Lebanon

## Abstract

*Introduction*. The aim of this study was to assess the complications resulting from third molar extraction under general anesthesia. *Material and Methods*. The retrospective study included all patients who underwent impacted third molars extraction from January 2008 until December 2011. 7659 third molars were extracted for 2112 patients. Postoperative complications were retrieved from medical files. *Results*. No complications were related to general anesthesia. The most frequent postoperative complication was infection (7.15%). Lingual nerve injuries affected 1.8% of the patients. All of them were transient and were not related to tooth section. Inferior alveolar nerve injuries were reported in 0.4% of the cases. 95.8% of these patients were admitted for one-day ambulatory care, and only two patients were readmitted after discharge from hospital. *Discussion*. This surgical technique offers comfort for both surgeons and patients. Risks are only linked to the surgical procedure as we observed no complication resulting from general anesthesia. One-day hospitalization offers a good balance between comfort, security, and cost. The incidence of complications is in agreement with the literature data, especially regarding pain, edema, and infectious and nervous complications. It is of utmost importance to discuss indications with patients, and to provide them with clear information.

## 1. Introduction

Postoperative complications following third molars surgical removal are a remaining topic of discussion with a fallacy of two schools of thought.

One school of thought is endorsed by oral and maxillofacial surgeons who contend that most third molars are potentially pathologic and should be removed. The other holds that only third molars with associated pathology should be removed.

The legal system, in which decisions are generally based on norms of practice or local or regional standards of care, credits each school of thought as having equal merit, ignoring the scientific evidence base [1].

A US National Institute of Health consensus conference meeting in 1979 attempted to establish wide agreement on the indications for third molar surgery. Many issues (including the role of surgical prophylaxis) were unresolved, although consensus was reached on factors which definitely merited surgery [2].

The British Association of Oral and Maxillofacial Surgeons (BAOMS), the Faculty of Dental Surgery at Queen Alexandra Hospital, Portsmouth, UK, and its Faculty Audit Working Group have written draft clinical guidelines to guide those involved clinicians [3].

Several published papers have raised issues which have implications not only for health service financing but also for the medico legal aspects of professional practice [4].

In the United States more than 11 million patient days of “standard discomfort or disability”—pain, swelling, bruising, and malaise—result from thirst molars extraction, and more than 11000 people sustained permanent paresthesia—numbness lip, numbness tongue, and cheek numbness—as a consequence of nerve injury during the surgery [1]. Surgical removal of impacted mandibular third molars should be carried out well before the age of 24 years, especially for female patients. Older patients are at greater risk of postoperative complications and permanent sequelae. A surgeon's lack of experience could also be a major factor in the development of postoperative complications [5].

The risk factors associated with complicationssuggest that age, tooth location, bone removal, and tooth sectioning appear to be associated with a higher complication rate for third molars removal [6, 7].

The extraction of third molars is one of the most common acts in dental practice and certainly the most frequent in stomatology. The indications for general anesthesia concerning this type of intervention are the subject of an ongoing debate, but the choice of the patient is critical and must be framed by clear information.

Like any surgical procedure, extraction of third molars under general anesthesia is not without complications either related to surgical intervention or to the type of anesthesia. Some complications may be subject to litigation and should be reported to the patient before his/her consent.

The aim of this work was to study postoperative complications associated with this type of intervention during four years of practice. The different postoperative complications have been reported with their proportions. The safety and comfort of general anesthesia during this period have been assessed in comparison with different studies in this field. Particular attention was given to the collection of all data concerning all complications such as those found in the medical record and visit controls.

## 2. Material and Methods

This retrospective monocentric study gathered all observations of patients undergoing an extraction of at least one third molar under general anesthesia over a period of almost four years (between January 2008 and December 2011). It involved 2112 patients, their ages ranged between 11 and 79 years, with a median age of 19.5. The sex ratio was 6 females for 4 males. Approvals of the ethical committee and the board of the hospital were required. The anonymity in the data collection was strictly respected. Inclusion and exclusion criteria were as follows.

- Inclusion Criteria:
- patient programmed for surgical removal of at least one third molar teeth under general anesthesia;
- patient must have given his/her informed and signed consent;
- patient must be insured or beneficiary of a health insurance plan;
- patient is available for 6 months of follow-up;
- patient under anesthesiologist supervision.
- Exclusion Criteria:
- patient over 18 years old and under judicial protection, under tutorship,
- patient (or legal representative) refuses to sign the consent,
- the patient is pregnant, parturient, or breastfeeding;
- addiction or chronic pain treated with morphine;
- contraindication for regional anesthesia (e. g., congenital or acquired coagulopathy, infection, allergy);
- contraindication to local anesthetics, general anesthetics and analgesics, difficult cooperation and psychiatric disorders that could interfere with assessments;
- contraindication for all states of sodium and water retention, especially heart failure, edema, and hydroperitoneum associated with cirrhosis.

Patients were referred by their treating dentist, orthodontist, or directly by the oral surgeon. In Less than 1% of cases, patients were referred by an oncologist in order to eliminate an infection in the distribution showed in Table 1.

The indications for extraction are listed in Table 2.

The indications in association with multiple extractions involved 11.5% of cases: patients with extensive dental decay problem but also patients candidate for a head and neck radiotherapy, chemotherapy, or bisphosphonates treatment.

Combination of third molars to maxillary or mandibular cysts was an indication for intervention under general anesthesia for 2% of patients. Finally, in less than 1% of cases, the complaint was part of a pain-dysfunction syndrome of the masticatory system. 

During preoperative consultation, we proposed and explained to all patients the two possible alternatives: intervention under local or locoregional analgesia and intervention under general anesthesia. Risks of oroantral fistulae (OAF) and labiomental dysesthesia were clearly exposed to patients with a potential higher risk.

An orthopantomogram was often sufficient in the radiological check-up. A Dentascan was performed whenever a root-nerve conflict was suspected.

All surgeries were carried out with the same surgical team, equipment, and technique. Surgical technique was classic: patient in supine position, nasal intubation, “packing Pharyngeal,” no periodontal infiltration, sulcular incision, and flap raising as of the first or second molar with subsequent discharge. Bone removal, dental cutting, and separation (cracking) were carried out using a single bur in tungsten carbide. Sutures required the use of braided polyglactin 910 suture (Vicryl Rapide 3/0.).

In some cases, an absorbable gelatin sponge (Gelfoam) was placed in alveolar cavity to stop bleeding in the sites of the upper third molars. Exceptionally, a sterile hemostatic gauze (Surgicel) was used. An intraoperative dose of 2 g of Amoxicillin or 600 mg of Clindamycin, if allergic to Penicillin, was administered as well as a dose of 1 mg/kg of Methyl prednisolone. Antibiotic treatment was continued postoperatively to all patients for a period of 2 to 6 days depending on the operating surgeon, along with an analgesic (Ibuprofen with 1200 mg dosage, divided in 2 or 3 intakes per day for a period of 3 to 5 days). Postoperative treatment and precautions were given to patient after surgery in Table 3.

7659 teeth were extracted of which 3731 maxillary teeth (1865 and 1866 right to left) and 3928 mandibular (1962 and 1966 right to left) exist, an average of 3.6 teeth per patient, ranging from a single tooth to six third molars! Eight patients had one supernumerary third molar while three patients had two. The third molars germs represented 43.9% of all extracted teeth.

The extraction of one third molar under general anesthesia has always been motivated by the need of another dental procedure such as multiple extractions, frenectomy, cyst excision, or surgical exposure of an impacted canine. The duration of the intervention, from incision to the “packing retrieval,” was around 33 minutes, with extremes going from a range of 10 to 90 minutes. This period also included actions such as associated additional extractions, exposure of canine, frenectomy, or resection of bone or mucosa lesions. The average occupancy of the operating room was 55 minutes. A Postoperative consultation was planned for all patients between the tenth and the fifteenth postoperative days.

## 3. Results

Out of the 2112 patients examined, 254 patients presented a complication with a minimum follow-up of 6 months (12%) Table 1. Postoperative abnormalities information was given to patient for normal and abnormal situations in Table 4.

No major incident was due to the type of anesthesia.

The infectious complications concerned 7.15% of our patients, a total of 151 patients. Complaints of pain or swelling were identified during the postoperative consultation or an emergency one. Pain motivated a consultation for 28 patients (1.3%) without the conclusion of an infection during examination.

38 patients, or 1.8% of cases, showed sensory disturbances of the labiomental area which was transient in all cases. The hemilateral section of the mandibular tooth concerned 20 of these patients with a rate of 52.6%, while in the other 18 cases (47.4%), there was no dental section. The degree of recovery was judged satisfactory by the patient, between the second and third postoperative months. Sensory disturbances of the labiomental area was detected in 9 patients (0.4% of all patients). In all cases, this disorder was hemi-lateral to sectioned tooth. Partial regression was experienced by the patient after 3 months in 6 cases, while in three other cases, it did not seem to improve during the same period. These 9 patients did not attend further consultations; therefore no other information is available on the recovery process.

Among other complications, we detected hemorrhage occurring between the first and the eighth days in the case of 6 patients. This required a revision under local analgesia including cleaning of the site and Gelfoam or Surgicel placement followed by tight suturing. Dysfunction of the masticatory system was observed in the case of 19 patients (0.9%): in 5 cases it concerned a limitation of mouth opening with musculoarticular pain evident in immediate postoperative period and resolved after 3 to 8 weeks.

In the case of 14 patients, a pain-dysfunction syndrome occurred during the months following the operation (between 3 months and 3 years). Delayed healing after closure of an oroantral fistula (OAF) was seen in the case of two patients. Spontaneous closure was observed in 3 months in the case of the first patient. The second patient, operated for a maxillary cyst adjacent to third molars, sustained maxillary sinusitis in the postoperative check-up. The fistula was closed 4 months and a half after surgery. Finally, the accidental mobilization of a sound second molar complicated the gesture in a single case.

However, the tooth was not lost. A following two-month control visit confirmed a grade 1 mobility (less than 1 mm). 95.8% of patients had a one-day hospitalization, and only 4.2% were kept for one night because of their medical history or postoperative state of health. We had to readmit two patients to the hospital: the first one for nausea and vomiting that occurred two days after the surgery and the second one for bleeding that started eight days after surgery without detection of any hematological cause. The cessation of any professional or scholar activities was for an average of a week.

## 4. Discussion

Infectious complications were the most common outcome of the surgery, reaching 7.15% of patients in this study despite a routine prescription of antibiotics. The reported rate is probably overestimated, since we considered as an infection all persistent or secondary swelling, mucosal inflammation, loosening of sutures, or pain implicating a second antibiotic prescription. The Anglo-Saxon term “inflammatory complications” would certainly have been more appropriate. This rate of 7.15% is close to the 8% shown in a French study conducted under the same conditions [8]. According to Trost and coworkers infectious complications vary between 2% and 13% in the literature [9, 10], which places our study results in the range of average.

Antibiotic prophylaxis/therapy for the extraction of third molars is a regular subject of controversy, although double-blinded randomized trials have concluded an important value to the use of it including Amoxicillin, Amoxicillin/Clavulanic acid, or Clindamycin [11–13].

The situation is much more delicate when it comes to lingual nerve or inferior alveolar nerve damages. We found a lingual hypoesthesia (always transient) in 1.8% of patients. The hemilateral tooth section procedure was not implicated in these cases. A sensory labiomental dysesthesia (anesthesia, hypoesthesia, or paresthesia) was reported in 0.4% of cases, always in relation with the hemilateral tooth section. We do not have sufficient data on their long-term evolution, even if partial regression was observed within 3 months after surgery in 6 of the 9 cases. Trost and coworkers [8] reported a rate of 1% for transient lingual hypoesthesia and 2% for labiomental hypoesthesia in a study including 180 patients. Blondeau and Daniel [5], in a prospective study with a decline of 24 months concerning 550 mandibular molars, reported 1.1% of sensory disorders related to a lesion of the inferior alveolar nerve and did not report any case, even transient, of lingual hypoesthesia. Jerjes and coworkers [14], in a prospective study including 3236 patients, related a sensory disturbance incidence to a lingual nerve damage (1.8%) and mandibular nerve damage (1.5%) a month after the intervention. Permanent deficits were estimated at 1.1% to the lingual nerve and 0.6% to the inferior alveolar nerve.

The sensitive-sensory deficit is associated with a lingual nerve injury causing more discomfort to the patient and would result in the most frequent medico legal litigation [14].

Damage of lingual nerve may inexplicably appear despite a well-controlled technique because of the multiple anatomical variations [15]. Therefore, a question surfaces: a random, rare, and unpredictable outcome should be a warning before surgery? Boffano and coworkers disagree with this idea, given the fact of the rarity of this complication and the additional amount of anxiety inflicted on the patients [15].

Asked to comment on the legal aspect of iatrogenic disorders, Lawton [16], a legal specialist in the UK, suggested that the most important legal problem facing the majority of practitioners is not negligence but obtaining the informed consent for the treatment. This consent is mandatory as soon as the risk of developing a serious complication or discomfort is considered “reasonable”. According to Lawton, the risk is real if it can affect one in ten patients, probably even if it is a patient in a hundred, but then chances decrease to get distant from reality and approach possibility, but the law is not preoccupied by the possibilities.

Cited by Boffano and coworkers [15], the American consensus conference on the extraction of third molars under the auspices of the US National Institutes of Health in 1979 recommended informing the patient of any potential risk higher or equal to 5% if transient and superior to 0.5% if permanent.

In the case of our patients, we always try to explain during the preoperative consultation the risks of labiomental sensory disorders and OAF whenever these risks are potentially present. We then mention it in the patient's medical record. But we judge it as unnecessary, in the light of our experience, to inform our patients on the risk of a lingual nerve disorder because of its rarity, the inability to predict it, and its transient nature in the majority of the cases. We stipulate it though in the postoperative information sheet as a possible postoperative complication. We are not fearing a rejection of the surgery by the patient, but trying to avoid additional stress to often anxious patients.

The extraction of third molars under general anesthesia is far from being qualified as an outdated operation. The majority of patients consulting our practice express a request for general anesthesia because of “fear or dental phobia.” Many authors have studied this issue, and it would appear that anxiety concerning dental care is observed worldwide, with variations according to age, sex, and experience [17–19]. This type of anesthesia provides comfort for both patients and practitioners without necessarily increasing the risk, when it is performed in good conditions [20]. The collaboration with the anesthesiologist in the operating room during the intervention (legal obligation in Belgium) and the standardization of surgical procedure considerably save time. The use of a single tungsten carbide bur to expose and separate the teeth avoids wasting time and therefore a longer exposure to anesthetics.

Over 95% of our patients were admitted to a one-day care hospital, which significantly helped reducing the cost of care provided in Belgium mainly by the National Institute of Health and Disability Insurance. The economic cost, taking, for example, the extraction of four third molars under general anesthesia, would be around 1000 Euros per patient, including hospitalization and all services related to medical care. On top of that, a social cost occurs since the cessation of professional and scholar activities is around a week.

Among our 2112 patients, we had to readmit two patients: the first one for nausea and vomiting that occurred two days after surgery and the second one for bleeding starting 8 days following surgery. Both incidents compared to the number of patients in our study are an acceptable risk (<1/1000); especially given the benign nature of the complications imposing readmission. Safety rules induced prolonged hospitalization when necessary (unsatisfactory health state, impossibility of returning to the emergency unit in case of serious trouble, and absence of an accompanying person). The patients were cleared out by the end of the day after the visit of the surgeon, the anesthesiologist, and the explanation of the different complications that may occur during the recovery period.

Another matter is the hypersensitivity of the seven (37,47); it seemed in any case a complaint was sent to us as surgeon in this direction. Perhaps the complaint was found among dentists. However, some patients had caries (37,47) like other teeth before avulsion DDS, and that required support from their dentist. In this study, we consulted regarded complications after avulsion of DDS. No devitalisation has been done, and this entity pains of 7 is not really highlighted.

## 5. Conclusion

With the ever-growing need for oral health, in orthodontics and Aesthetics on one hand, and the public demand for suppression of pain or even discomfort on the other, it is reasonable to assume that surgical extraction of third molars under general anesthesia is an interesting intervention that retains its place in the practice of oral and maxillofacial surgery despite many controversies that this operation rises. This procedure should be framed by clear information, listing all the possible options including surgical abstention helping therefore the patient in making a decision. The gesture is not without complications; the majorly serious ones are fortunately rare and not dependent on the type of anesthesia, although there are “theoretical” risks during general anesthesia as well as any local or locoregional anesthesia. Surgical risk should be clearly set out without creating additional unnecessary stress.

## Figures and Tables

**Table 1:** Referral source.

Referral from	Percentage of patients
Dentist	45%
Orthodontist	36%
Surgeon (directly)	18%
Oncologist	<1%

**Table 2:** Indications for extraction of the third molars.

Indication for extraction	Percentage of patients
(1) Orthodontic reason (potential or existing crowding)	57%
(2) Painful tooth A + B	28.5%
(A) Abscess or pericoronitis related to the eruption of third molars (infection)	8.5%
(B) Episodes of recurring discomfort or “tightening” pain type without proven infection	20%
(3) Patients with extensive dental decay problem but also patients candidates for a head and neck radiotherapy, chemotherapy, or bisphosphonates treatment	11.5%
(4) Combination of third molars to maxillary or mandibular cysts was an indication for intervention under general anesthesia	2%
(5) Complaint was part of a pain-dysfunction syndrome of the masticatory system	1%

**Table 3:** Postoperative recommendations distributed to patient after surgery.

Postoperative treatment	(1) Amoxicillin 1 g: 1 tablet in the morning and one in the evening (during 4 days). (2) Ibuprofen 600 mg: 1 tablet in the morning, one at noon, and one in the evening, for 4-5 days (3) Acetaminophen 1 g in case of pain (maximum 4 times per day) (4) Mouth rinsing: to start the day after the surgery. After each meal: dilute a tablespoon in a glass of water. To do for 15 days.

Postoperative precautions	(i) Apply ice packs immediately to all areas where surgery was performed. Place ice packs on for 30, then off for 30 minutes. Continue at least 24–48 hours (ii) soft diet (iii) Position = ideal semisitting (avoid supine position) (iv) No effort or stress for 2 days. Avoid all excessive activity.

**Table 4:** Post operative abnormalities information given to patient after surgery.

Normal postoperative	(i) Swelling will peak 2 days after surgery (ii) Slow deflation during the next 5 days (iii) Difficulty and limitation in mouth opening (swelling) (iv) Appearance of a hematoma on the fourth day, after the deflation (v) Moderate pain resolving by medication intake (vi) Taste of blood with a slight discharge for 2-3 days (vii) Sutures annoying in the cheek and between the teeth (viii) Sensation that the teeth are sutured to the cheek (ix) Numbness of tongue (usually one week, but can last longer) (x) Sutures are absorbable

Postoperative abnormalities	Reinflation after the starting of deflation (rebound edema) (i) Important bleeding = hemorrhage. What to do: sit down; bite on gauze on the side that bleeds for 30 minutes with ice on the cheek. Check the bleeding after 30 minutes, and if bleeding continues, contact us. (ii) Temperature >38°C In case of problems, contact us
